# Hydration Strategies for Preventing Contrast-Induced Acute Kidney Injury: A Systematic Review and Bayesian Network Meta-Analysis

**DOI:** 10.1155/2020/7292675

**Published:** 2020-02-11

**Authors:** Qiuping Cai, Ran Jing, Wanfen Zhang, Yushang Tang, Xiaoping Li, Tongqiang Liu

**Affiliations:** Division of Nephrology, The Affiliated Changzhou NO.2 People's Hospital of Nanjing Medical University, Changzhou 213003, Jiangsu, China

## Abstract

**Aims:**

Many previous studies have examined the effect of different hydration strategies on prevention of contrast-induced acute kidney injury (CI-AKI), but the optimal strategy is unknown. We performed a network meta-analysis (NWM) of these previous studies to identify the optimal strategy.

**Methods and Results:**

Web of Science, PubMed, OVID Medline, and Cochrane Library were searched from their inception dates to September 30, 2018. Randomized controlled trials (RCTs) were selected based on strict inclusion criteria, and a Bayesian NWM was performed using WinBUGS V.1.4.3. We finally analyzed 60 eligible RCTs, which examined 21,293 patients and 2232 CI-AKI events. Compared to intravenous 0.9% sodium chloride (reference), intravenous sodium bicarbonate (OR [95% CI]: 0.74 [0.57, 0.93]), hemodynamic guided hydration (0.41 [0.18, 0.93]), and RenalGuard guided hydration (0.32 [0.14, 0.70]) significantly reduced the occurrence of CI-AKI. Oral hydration and intravenous 0.9% sodium chloride were each noninferior to no hydration in preventing CI-AKI. Intravenous 0.9% sodium chloride, sodium bicarbonate, and hemodynamic guided hydration were each noninferior to oral hydration in preventing CI-AKI. Based on surface under the cumulative ranking curve values, the RenalGuard system was best (0.974) and hemodynamic guided hydration was second best (0.849).

**Conclusion:**

There was substantial evidence to support the use of RenalGuard or hemodynamic guided hydration for preventing CI-AKI in high-risk patients, especially those with chronic kidney disease or cardiac dysfunction.

## 1. Introduction

Contrast-induced acute kidney injury (CI-AKI), also referred to as contrast-induced nephropathy (CIN), is an iatrogenic complication that can occur following intravascular administration of iodinated contrast medium (CM) prior to radiography. CI-AKI is the third leading cause of hospital-acquired acute renal injury (AKI) [1]. CI-AKI has a low incidence in the general population, but it has a significant incidence in patients with certain risk factors. Moreover, the occurrence of CI-AKI following cardiac catheterization procedures is associated with an in-hospital mortality of 20%, a 1-year mortality of up to 66%, and an even higher mortality in patients who require dialysis [2, 3]. However, even if patients with high risk of CI-AKI can be identified a priori, no known pharmaceutical treatment can effectively prevent or treat CI-AKI.

Guidelines recommend intravascular hydration to prevent CI-AKI [4, 5], and there are several specific hydration strategies, but researchers have not yet established an optimal strategy [6–9]. Notably, recent randomized controlled trials (RCTs) have led to doubts about the effectiveness of various hydration strategies in prevention of CI-AKI. For example, Nijssen et al. [10] conducted an RCT with 660 high-risk patients and found that no prophylaxis was noninferior or cost-saving relative to intravenous hydration. Weisbord et al. [11] enrolled 5177 high-risk patients and reported no benefit of intravenous sodium bicarbonate relative to normal saline. Another RCT [12] concluded that the benefit of sodium bicarbonate was marginal relative to isotonic sodium chloride for preventing CI-AKI among critically ill patients. However, other studies indicated that the RenalGuard System [13–16] and hemodynamic guided hydration [17–19] were safe and effective in preventing CI-AKI. Because of these apparently discrepant results, we conducted a network meta-analysis (NMA) to assess the effects of various hydration strategies on the occurrence of CI-AKI in an effort to identify the optimal strategy for prevention of CI-AKI.

## 2. Methods

### 2.1. Data Search

This systematic review and meta-analysis were performed according to Cochrane Handbook guidelines [20]. The Web of Science, PubMed, OVID Medline, and Cochrane Library databases were searched using medical subject headings or keywords. Relevant published original studies that were published up to September 30, 2018, were examined. The search syntax was as follows: “contrast-induced acute kidney injury OR contrast-induced nephropathy OR CIN OR CI-AKI OR contrast acute renal failure OR contrast nephropathy” AND “hydration OR fluid administration OR volume expansion OR intravenous sodium bicarbonate OR saline infusion.”

### 2.2. Study Selection

An initial eligibility screen of all citations was conducted, and only studies that examined CI-AKI and hydration were selected for further full-text review. All included studies were RCTs; experimental studies were excluded. In addition, all included studies reported the prevention of CI-AKI after intravascular administration of CM; used clinical protocols that were hydration strategies, not pharmaceutical prevention strategies; had clear definitions of CI-AKI; and provided data on the outcome of interest (occurrence of CI-AKI within 2 days to 1 week after procedures).

### 2.3. Data Extraction and Quality Assessment

Two authors (C. Q. P. and J. R.) independently reviewed each article for eligibility. Any disagreement was resolved by discussion among the authors or involvement of a third author. Data extraction included the year of publication, sample size, patient characteristics, risk factors associated with CI-AKI (old age, diabetes mellitus, renal impairment, heart failure), and type and dosage of contrast medium. The primary endpoint was the occurrence of CI-AKI within 2 days to 1 week after intravascular administration of CM. Two investigators independently evaluated the quality of each study using the Jadad scale, which ranges from 0 (worst) to 5 (best) [21].

### 2.4. Statistical Analyses

The advantages of Bayesian NMA over traditional meta-analysis are its greater flexibility, its provision of more naturally interpretable results, and its ability to rank treatments by comparative effectiveness [22]. The occurrence of CI-AKI as a dichotomous outcome variable was expressed as an odds ratio (OR) and 95% confidence interval (CI). All *P* values were 2-sided, and a *P* value below 0.05 was considered significant. All analyses were conducted using the Bayesian Markov chain Monte Carlo method in WinBUGS V.1.4.3 (MRC Biostatistics Unit, Cambridge, United Kingdom) using the Microsoft Excel-based macro NetMetaXL V.1.6.1 (Canadian Agency for Drugs and Technologies in Health, Ottawa, Canada) [23]. A convergence test for each analysis was conducted by checking whether the Monte Carlo error was less than 5% of the SD of the effect estimates or the variance between the studies. Convergence was achieved for all analyses using 1000 “burn in” runs and 1000 model runs. NetMetaXL was also used to generate a forest plot, league table, and “rankogram” with surface under the cumulative ranking curve (SUCRA), which ranges from 0 (worst) to 100% (best). Inconsistency was assessed by comparing the residual deviance and deviance information criterion statistics in fitted consistency and inconsistency models.

## 3. Results

### 3.1. Literature Search

**Figure 1:**
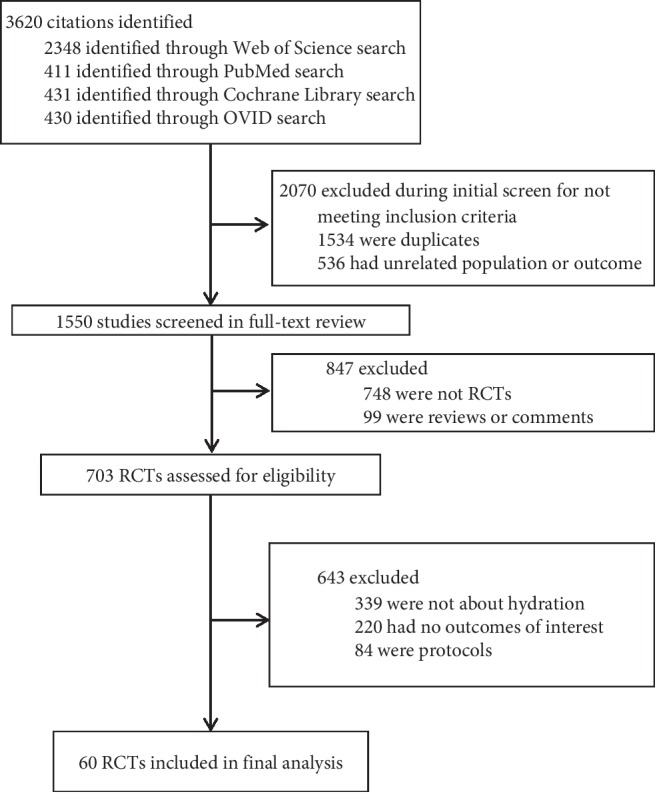
Identification and selection of studies for Bayesian network meta-analysis.

We initially identified 3620 publications, assessed 703 RCTs for eligibility by review of the full texts, and ultimately included 60 RCTs which met the eligibility criteria (Figure 1). These studies examined 21,293 patients (median: 222, interquartile range [IQR]: 120, 350) and 2232 CI-AKI events. All included RCTs were full-length journal articles. Agreement between the two reviewers at the full-text review stage was excellent (Cohen's *κ* = 0.85).

### 3.2. Characteristics of Studies and Participants

Table 1 shows the characteristics of the included studies. The publication date ranged from 2002 to 2018, and about 50% of the studies were published after 2013. The proportion of male patients ranged from 25.0% to 98.1% (median [IQR]: 65.7 [56.9, 74.8]), and the mean age ranged from 56.2 to 82.9 years (67.8 [63.1, 72.5]). Thirty-one studies enrolled 12,519 patients who had high risk of CI-AKI. The baseline serum creatinine (SCr) level ranged from 61.4 to 236.4 *μ*mol/L (117.1 [89.5, 136.9]), and the baseline estimated glomerular filtration rate (eGFR) ranged from 32 to 93.1 mL/min/1.73 m^2^ (49.2 [44.1, 74.2]). Twenty-three studies provided the values of left ventricular ejection fraction (LVEF); the mean LVEF ranged from 25% to 57.8% (49.0 [42.8, 54.5]). The percentage of diabetes mellitus (DM) patients ranged from 8% to 100%, and the percentage with heart failure (HF) ranged from 0.6% to 45.8%. A total of 8176 patients from 32 studies received intravenous low-osmolar nonionic CM, 9993 patients from 17 studies received iso-osmolar nonionic CM, and 317 patients from 2 studies received low-osmolar ionic CM. The mean Jadad score of the 60 RCTs was 3.2 (3 [2, 4]), indicating the overall study quality was good.

### 3.3. Network Meta-Analysis

**Figure 2:**
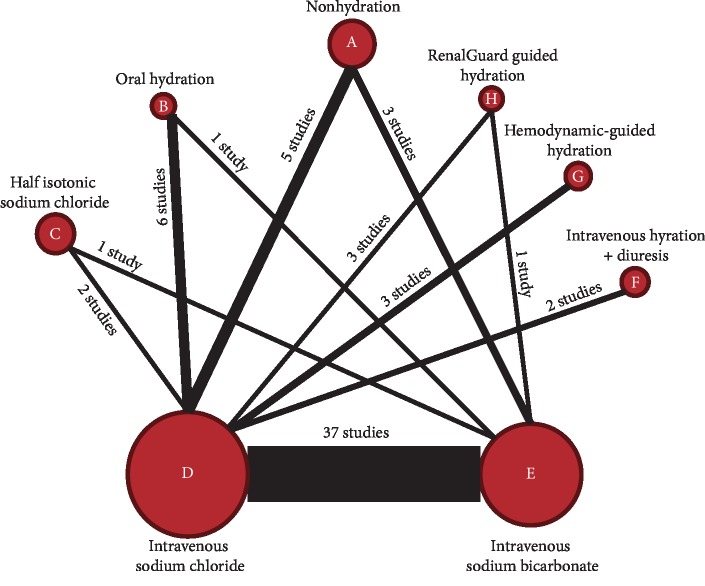
Network diagram of eight hydration strategies used to prevent contrast-induced acute kidney injury in the 60 included studies. Circles represent hydration strategies and lines represent direct comparisons. Circle size indicates the number of participants who received each treatment, and line thickness indicates the number of studies in each comparison.

Figure 2 shows all the comparisons in the NMA. Thirty-seven studies (13,365 participants) compared the efficacy of intravenous sodium bicarbonate and 0.9% sodium chloride. The other hydration strategies were nonhydration (8 studies, 1396 patients), oral hydration (6 studies, 355 patients), intravenous half iso-osmolar saline (3 studies, 968 patients), intravenous hydration, mainly normal saline + diuresis (2 studies [26, 31], 501 patients), hemodynamic guided hydration (3 studies, 458 patients), and RenalGuard system guided hydration (4 studies, 348 patients).

**Figure 3:**
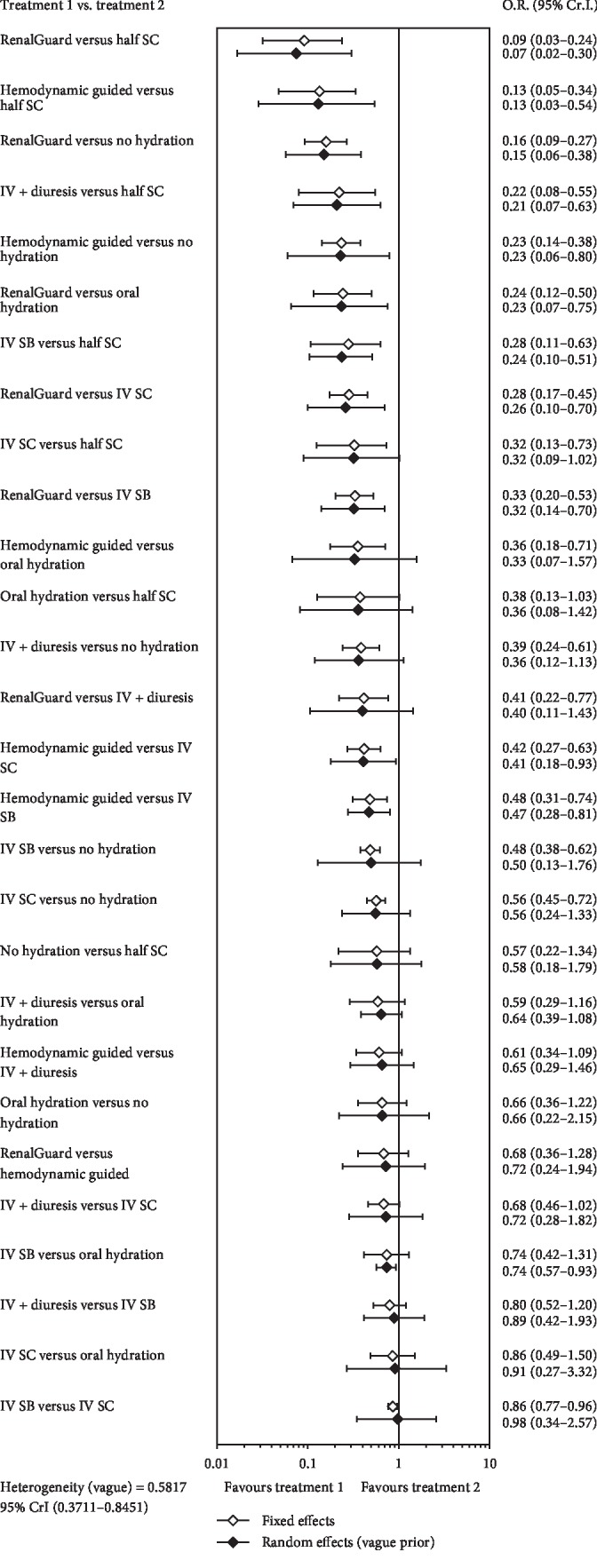
Forest plot showing the effect of different hydration strategies. Summary estimates from the pooled studies with 95% confidence intervals are indicated for fixed effects (open diamonds) and random effects (filled diamonds) models.

**Figure 4:**
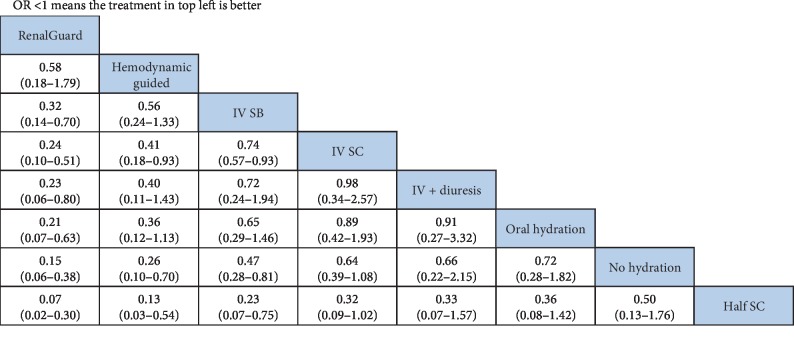
League table, showing all pairwise comparisons of studies.

We compared the ORs of the different hydration strategies using a forest plot (Figure 3) and analyzed the results of the random effects consistency NMA using a league table, which shows all pairwise comparisons (Figure 4). Taken together, these results indicate that, relative to typical intravenous 0.9% sodium chloride hydration (reference), the occurrence of CI-AKI was significantly reduced by intravenous sodium bicarbonate (OR [95% CI]: 0.74 [0.57, 0.93]), hemodynamic guided hydration (0.41 [0.18, 0.93]), and RenalGuard system guided hydration (0.32 [0.14, 0.70]). Oral hydration (0.72 [0.28, 1.82]) and intravenous 0.9% sodium chloride (0.64 [0.39, 1.08]) were each noninferior to no hydration for prevention of CI-AKI. Relative to oral hydration (reference), intravenous 0.9% sodium chloride or sodium bicarbonate and hemodynamic guided hydration were each noninferior in prevention of CI-AKI, but RenalGuard guided hydration was superior (0.21 [0.07, 0.63]). Intravenous hydration plus diuresis also did not decrease the risk of CI-AKI relative to oral hydration and no hydration.

**Figure 5:**
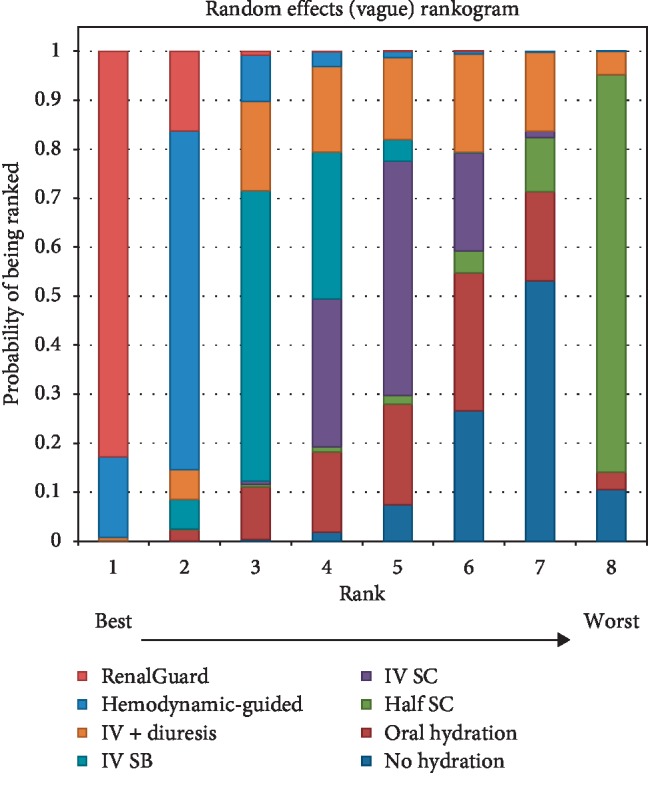
Rankogram of the effect of different hydration strategies in reducing the risk of contrast-induced acute kidney injury.

A rankogram and SUCRA values indicated the RenalGuard system was best (SUCRA = 0.974) followed by hemodynamic guided hydration (SUCRAs = 0.849; Figure 5). Intravenous sodium bicarbonate had a SUCRA of 0.667. The SUCRAs for intravenous 0.9% sodium chloride, intravenous hydration plus diuresis, oral and no hydration, and the other treatments ranged from 0.197 to 0.441, and their rankings were similar. Hydration using half iso-osmolar saline alone was the least effective treatment.

### 3.4. Inconsistency Analysis

**Figure 6:**
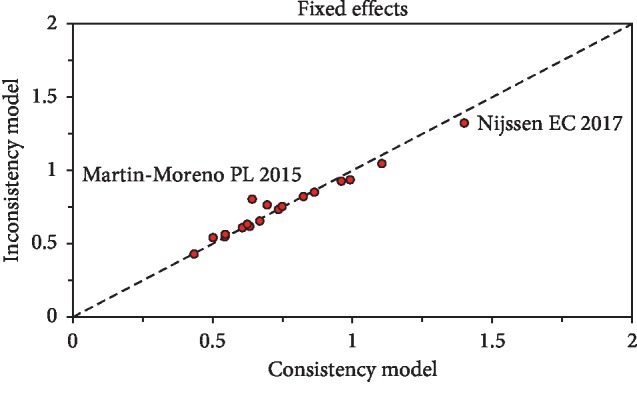
Inconsistency plot of enrolled studies, showing the posterior mean deviance of each study from the consistency model (horizontal axis) and the inconsistency model (vertical axis).

We performed network inconsistency assessment for the fixed effect model for the 60 studies (Figure 6). The resulting plot demonstrated that nearly all the studies were near the line of equality and that the results were therefore consistent. However, there was some evidence of inconsistency in 3 noninferiority studies [10, 31]. In particular, Martin-Moreno et al. [31] and Nijssen et al. [10] found that intravenous sodium bicarbonate and 0.9% sodium chloride were noninferior to oral hydration.

## 4. Discussion

To our knowledge, this is the first NMA to compare different hydration strategies for prevention of CI-AKI. We included 60 RCTs which examined 21,293 participants and 2232 CI-AKI events. Our comparison of 8 hydration strategies for preventing CI-AKI confirmed that, relative to intravenous 0.9% sodium chloride hydration, three treatments during CM administration significantly reduced the risk for CI-AKI: the RenalGuard system, hemodynamic guided hydration, and intravenous sodium bicarbonate. Relative to no hydration, oral hydration and intravenous 0.9% sodium chloride were each noninferior in prevention of CI-AKI. Relative to oral hydration, intravenous 0.9% sodium chloride and sodium bicarbonate were each noninferior in prevention of CI-AKI. Thus, we ranked the RenalGuard system as the best strategy and hemodynamic guided hydration as the second best.

Guidelines for the prevention of CI-AKI in high-risk patients routinely recommend hydration protocols before contrast exposure as an established preventive measure [77, 78]. A recent large RCT [10] led us to reanalyze the efficacy of hydration for prevention of CI-AKI. In particular, the AMAstricht Contrast-Induced Nephropathy Guideline (AMACING) study [10] enrolled 660 patients with high risk of CI-AKI and concluded that, relative to intravenous hydration, no prophylaxis was less expensive and noninferior in prevention of CI-AKI. In our meta-analysis, five studies compared the effectiveness of intravenous 0.9% sodium chloride and three studies compared bicarbonate with nonhydration, leading to our conclusion that, relative to no hydration (reference), oral hydration or hydration with intravenous 0.9% sodium chloride was noninferior in prevention of CI-AKI. These results were unsurprising, because simple oral or intravenous hydration can lead to complications, such as heart failure, pulmonary edema, and electrolyte disorders. Thus, the safety window of hydration is relatively narrow for patients undergoing percutaneous coronary intervention (PCI), and other more effective or precise hydration strategies may be needed to decrease the incidence of CI-AKI.

Most meta-analyses before 2016 [79–83] confirmed that intravenous sodium bicarbonate was more effective than sodium chloride in preventing CI-AKI. However, two recent influential studies concluded that intravenous sodium bicarbonate provided no benefit over intravenous sodium chloride in high-risk patients [11] and critically ill patients [12]. Our NMA included 37 studies that compared intravenous sodium chloride with sodium bicarbonate, and our results also indicated that intravenous sodium bicarbonate led to a reduced risk for CI-AKI, although the effect size was small (OR [95% CI]: 0.74 [0.57, 0.93]). Alkalization with bicarbonate perfusion could theoretically reduce the formation of reactive oxygen species by decreasing the production of hydroxyl radicals due to inhibition of the Haber-Weiss and Fenton reactions [84]. However, the HYDRAREA study [12] assessed 307 critically ill patients with stable renal function and found that hydration with bicarbonate provided no benefit relative to hydration with isotonic sodium chloride. These researchers also noted that bicarbonate provided a greater benefit in the smaller studies, suggesting publication bias. Recently, Weisbord et al. [11] enrolled 5177 patients with high risk for renal complications and found that administration of sodium bicarbonate did not reduce the occurrence of CI-AKI. This result supports the interpretation that sodium bicarbonate is not more effective than sodium chloride in preventing CI-AKI or longer-term adverse outcomes after angiography. However, there was high heterogeneity among our 60 studies regarding concurrent medications, comorbidities (CHF, DM), types of CM, periprocedural hydration protocols, concentrations and dosages of sodium bicarbonate, and radiographic procedures [12]. Thus, we do not recommend alkalization with intravenous sodium bicarbonate as a single strategy, and a more effective hydration strategy is needed to prevent CI-AKI.

Several recent RCTs of high risk patients [13, 28, 47, 55] showed that furosemide-induced high-volume forced diuresis with matched hydration using the RenalGuard system effectively prevented CI-AKI. RenalGuard is a closed-loop fluid-management system, in which each volume of urine that enters the collection bag leads to the infusion of an equal volume of saline into the patient. Two meta-analyses [14, 16] of RCTs concluded that the RenalGuard system significantly reduced the risk of CI-AKI and the need for renal replacement therapy in high-risk patients undergoing coronary angiography. Our rankogram analysis indicated that the RenalGuard system of guided hydration had the highest rank, with a SUCRA of 0.974. However, we did not assess the effectiveness of intravenous hydration plus diuresis without a guided system, and the rankogram indicated that hemodynamic guided hydration was the second best method, with a SUCRA of 0.849. Brar et al. [17] used left ventricular end-diastolic pressure to guide fluid administration and demonstrated that this method was safe and effective in prevention of CI-AKI among patients undergoing cardiac catheterization. Another study [19] demonstrated that central venous pressure-guided fluid administration safely and effectively reduced the risk of CI-AKI in patients with CKD and CHF. Maioli et al. [18] assessed body fluid level using bioimpedance vector analysis (BIVA), which allows adjustment of intravascular volume expansion, and this led to a lower incidence of CI-AKI after angiographic procedures. Therefore, our results indicate that the RenalGuard system and hemodynamic guided hydration are best for patients with high-risk for CI-AKI, especially those with CKD and cardiac dysfunction.

## 5. Limitations

It is essential to note several limitations of our study. Firstly, the hydration protocol should have a substantial influence on CI-AKI, but because of the high heterogeneity of specific protocols used in the included studies, we could not analyze distinct protocols, such as the effect of different concentrations of sodium bicarbonate, and the effect of hydration duration. Secondly, several confounding factors that we did consider may have impacted the effects of hydration, including dosage and types of CM, risk status of patients for CI-AKI, and other factors. Finally, it may be inappropriate to define hemodynamic guided hydration based on the use of different indexes, such as left ventricular end-diastolic pressure, central venous pressure, and bioimpedance.

## 6. Conclusion

This Bayesian NMA provided substantial evidence to support the use of RenalGuard or hemodynamic guided hydration to prevent CI-AKI in high-risk patients, especially those with CKD or cardiac dysfunction.

## Figures and Tables

**Table 1:** Characteristics of the included studies.

Studies	Patients (*n*)	Inclusion criteria/risk of CI-AKI	Mean age (years)	Males (%)	Baseline SCr (mg/dL)	Baseline eGFR (mL/min/1.73 m^2^)	Mean LVEF (%)	DM (%)	HF (%)	Treatment groups	Types of CM	CM dosage (mL)	Jadad score	No. of patients	Inclusion criteria/risk of CI-AKI	Mean age	Male (%)	Baseline SCr	Baseline eGFR	Mean LVEF	DM (%)	HF (%)	Groups	Types of CM	Dosage of CM	Jadad score
Weisbord et al. [11]	4993	High risk for renal complications and scheduled for angiography	69.8	93.6	132.6	50.2		80.9	7.4	SC vs. SB	Iodixanol or low-osmolar	85	5	4993	High risk for renal complications and scheduled for angiography	69.8	93.6	132.6	50.2		80.9	7.4	SC vs. SB	Iodixanol or low-osmolar	85	5

van Mourik et al. [24]	74	Symptomatic aortic valve stenosis and impaired renal function who underwent pre-TAVI CTA	82.9	44.6	104.3	47.4		31.1		SC vs. SB	Iopromide	90	3	74	Symptomatic aortic valve stenosis and impaired renal function who underwent pre-TAVI CTA	82.9	44.6	104.3	47.4		31.1		SC vs SB	Iopromide	90	3

Saratzis et al. [25]	58	Elective EVAR for infrarenal AAA	75	89.7		65.5		13.8		SC vs. SB	Iomeprol	126	3	58	Elective EVAR for infrarenal AAA	75	89.7		65.5		13.8		SC vs SB	Iomeprol	126	3

Maioli et al. [18]	296	Elective coronary angiographic procedures	71	68.2	89.3		48	24.7		SC vs. HDy	Iodixanol	131	3	296	Elective coronary angiographic procedures	71	68.2	89.3		48	24.7		SC vs HDy	Iodixanol	131	3

Kooiman et al. [26]	333	CKD patients undergoing elective cardiovascular diagnostic or interventional contrast procedures	73	64.6		50.5		38.7	16.5	SC vs. SB	Not mentioned	113	3	333	CKD patients undergoing elective cardiovascular diagnostic or interventional contrast procedures	73	64.6		50.5		38.7	16.5	SC vs SB	Not mentioned	113	3

Valette et al. [12]	307	Critically ill patients with stable renal function who received intravascular CM	56.2	67.8	61.4			13.4	6.5	SC vs. SB	Low-osmolar	90	4	307	Critically ill patients with stable renal function who received intravascular CM	56.2	67.8	61.4			13.4	6.5	SC vs SB	Low-osmolar	90	4

Nijssen et al. [10]	660	High-risk patients with eGFR of 30–59 ml/min/1.73 m^2^, undergoing an elective procedure requiring CM administration	72	61.7	118	47.4		32.6		Non vs. SC	Iopromide	90.5	3	660	High-risk patients with eGFR of 30–59 ml/min/1.73 m^2^, undergoing an elective procedure requiring CM administration	72	61.7	118	47.4		32.6		Non vs SC	Iopromide	90.5	3

Alonso et al. [27]	93	Patients receiving CM during CRT devices implantation	66.5	65.3	110.5		28.5	37		SC vs. SB	Iodixanol	102	2	93	Patients receiving CM during CRT devices implantation	66.5	65.3	110.5		28.5	37		SC vs SB	Iodixanol	102	2

Usmiani et al. [28]	124	Coronary angiography/PCI with eGFR of less than 60 ml/min/1.73 m^2^	75	74	130.8	44	25	84		SC vs. RenalGuard	Iodixanol	156	3	124	Coronary angiography/PCI with eGFR of less than 60 ml/min/1.73 m^2^	75	74	130.8	44	25	84		SC vs RenalGuard	Iodixanol	156	3

Turedi et al. [29]	172	Contrast-enhanced CTPA on suspicion of PE with at least one risk factor for CIN	75.5	51.7	85.4					SC vs. SB	Water-soluble, nonionic, low-osmolar	<100	3	172	Contrast-enhanced CTPA on suspicion of PE with at least one risk factor for CIN	75.5	51.7	85.4					SC vs SB	Water-soluble, nonionic, low-osmolar	<100	3

Qian et al. [19]	264	CKD and CHF undergoing coronary procedures	63.5	74.6	151	37.5	39.5	47.3		SC vs. HDy	Iodixanol	166	5	264	CKD and CHF undergoing coronary procedures	63.5	74.6	151	37.5	39.5	47.3		SC vs HDy	Iodixanol	166	5

Solomon et al. [30]	391	Elective coronary or peripheral angiography with eGFR <45 ml/min/1.73 m^2^	72	57.5	169.3	32.8		59.1	35.5	SC vs. SB	Not mentioned	107	4	391	Elective coronary or peripheral angiography with eGFR <45 ml/min/1.73 m^2^	72	57.5	169.3	32.8		59.1	35.5	SC vs SB	Not mentioned	107	4

Martin-Moreno et al. [31]	130	Receiving CM for CT scan	57.5	64.3	79.6					Non vs SB	Not mentioned	120	3	130	Receiving CM for CT scan	57.5	64.3	79.6					Non vs SB	Not mentioned	120	3

Jurado-Román et al. [32]	408	STEMI undergoing primary PCI	63.1	73.4		89		22.5	14.7	Non vs. SC	Iso-osmolar nonionic	174	2	408	STEMI undergoing primary PCI	63.1	73.4		89		22.5	14.7	Non vs SC	Iso-osmolar nonionic	174	2

Barbanti et al. [13]	112	TAVR	81	40.2	87.1	51.5	54.6	25		SC vs. RenalGuard	Buckinghamshire	175	3	112	TAVR	81	40.2	87.1	51.5	54.6	25		SC vs RenalGuard	Buckinghamshire	175	3

Yeganehkhah et al. [33]	100	CAG	59.7	53	99.5		43.8	39		SC vs. SB	Iohexol	45.4	3	100	CAG	59.7	53	99.5		43.8	39		SC vs SB	Iohexol	45.4	3

Yang et al. [34]	320	Elective cardiovascular procedures including CAG or interventional treatment	59.2	53.1	70.2	93.1	55.1	20		SC + NAC vs. SB + NAC	Iopromide	125	3	320	Elective cardiovascular procedures including CAG or interventional treatment	59.2	53.1	70.2	93.1	55.1	20		SC + NAC vs SB + NAC	Iopromide	125	3

Yang et al. [34]	320	Elective cardiovascular procedures including CAG or interventional treatment	59.2	53.1	70.2	93.1	55.1	20		SC vs. SB	Iopromide	125	3	320	Elective cardiovascular procedures including CAG or interventional treatment	59.2	53.1	70.2	93.1	55.1	20		SC vs SB	Iopromide	125	3

Thayssen et al. [35]	362	STEMI undergoing primary PCI within 12 hours from the onset of chest pain	62.5	78.5	77	90.5	50	9.7		SC vs. SB	Iodixanol	140	5	362	STEMI undergoing primary PCI within 12 hours from the onset of chest pain	62.5	78.5	77	90.5	50	9.7		SC vs SB	Iodixanol	140	5

Nieto-Rios et al. [36]	220	Tomography scan using CM or angiography	60	57.7	115.8			37.3		SC vs. SB	Iohexol	100	3	220	Tomography scan using CM or angiography	60	57.7	115.8			37.3		SC vs SB	Iohexol	100	3

Manari et al. [37]	592	STEMI within 12 h from symptom onset referred for primary angioplasty	65	74.8	88.5	81	48	16.6	11.8	SC vs. SB	Iodixanol	198	3	592	STEMI within 12 h from symptom onset referred for primary angioplasty	65	74.8	88.5	81	48	16.6	11.8	SC vs SB	Iodixanol	198	3

Mahmoodi et al. [38]	350	Coronary interventions	64.48	51.4	103	64.8				SC vs. SB	Iohexol		2	350	Coronary interventions	64.48	51.4	103	64.8				SC vs SB	Iohexol		2

Luo et al. [39]	216	STEMI	67	65.7	77	77.6		25		Non vs. SC	Iopamiron	234.9	3	216	STEMI	67	65.7	77	77.6		25		Non vs SC	Iopamiron	234.9	3

Kooiman et al. [40]	548	CKD patients receiving CE-CT	72.1	60.4		50.4		26.8	16.4	SC vs. SB	Iomeprol	105	5	548	CKD patients receiving CE-CT	72.1	60.4		50.4		26.8	16.4	SC vs SB	Iomeprol	105	5

Kooiman et al. [41]	138	CKD patients receiving CTPA	70.5	50		49.2		16.7	8	Non vs. SB	Iopromide or iobitridol or iodixanol	74	5	138	CKD patients receiving CTPA	70.5	50		49.2		16.7	8	Non vs SB	Iopromide, or iobitridol, or iodixanol	74	5

Brar et al. [17]	396	Referred to the cardiac catheterization laboratory with eGFR ≤ 60 mL/min/1.73 m^2^, and at least one of the following: DM, CHF, hypertension, or age older than 75 years	72	61.9	123.8	48		51.3	20.5	SC vs. HDy	Ioxilan	108	3	396	Referred to the cardiac catheterization laboratory with eGFR ≤ 60 mL/min/1.73 m^2^, and at least one of the following: DM, CHF, hypertension, or age older than 75 years	72	61.9	123.8	48		51.3	20.5	SC vs HDy	Ioxilan	108	3

Akyuz et al. [42]	225	At least one of the high-risk factors for developing CI-AKI and undergoing CAG and/or PCI	63.4	68.9	79.6	84.5	47.5	60.9	7.6	Oral vs. SC	Not mentioned	108	2	225	At least one of the high-risk factors for developing CI-AKI and undergoing CAG and/or PCI	63.4	68.9	79.6	84.5	47.5	60.9	7.6	Oral vs SC	Not mentioned	108	2

Kristeller et al. [43]	92	Stage 3 or higher CKD who underwent cardiac surgery using CPB	72.5	57.6	119.1			44.6	34.8	SC vs SB	Not mentioned	79	5	92	Stage 3 or higher CKD who underwent cardiac surgery using CPB	72.5	57.6	119.1			44.6	34.8	SC vs SB	Not mentioned	79	5

Koc et al. [44]	195	DM patients	62	52.3	88.4			100		SC vs. SB	Not mentioned	90	4	195	DM patients	62	52.3	88.4			100		SC vs SB	Not mentioned	90	4

Gu et al. [45]	859	Coronary angiography or angioplasty	59	72.2	90.1	74.2		20.6	0.6	SC vs. SC + diuresis	Not mentioned	100	2	859	Coronary angiography or angioplasty	59	72.2	90.1	74.2		20.6	0.6	SC vs SC + diuresis	Not mentioned	100	2

Boucek et al. [46]	120	Diabetic patients with impaired renal function, undergoing intra-arterial or intravenous use of CM	65	75	165	44.1		100		SC vs. SB	Low-osmolar nonionic iodinated	110	5	120	Diabetic patients with impaired renal function, undergoing intra-arterial or intravenous use of CM	65	75	165	44.1		100		SC vs SB	Low-osmolar nonionic iodinated	110	5

Marenzi et al. [47]	170	CKD undergoing coronary procedures	73	78.2	154.7	39	51.5	36.4		SC vs. RenalGuard	Iomeprol	170	3	170	CKD undergoing coronary procedures	73	78.2	154.7	39	51.5	36.4		SC vs RenalGuard	Iomeprol	170	3

Kong et al. [48]	80	Definitive or suspected coronary artery disease	56.5	53.8	105			23.8		Oral vs. SC	Iopromide	152	3	80	Definitive or suspected coronary artery disease	56.5	53.8	105			23.8		Oral vs SC	Iopromide	152	3

Klima et al. [49]	258	Renal insufficiency undergoing intravascular contrast procedures	77	64	137	43.6		37	44	SC vs. SB	Not mentioned	100	5	258	Renal insufficiency undergoing intravascular contrast procedures	77	64	137	43.6		37	44	SC vs SB	Not mentioned	100	5

Gomes et al. [50]	301	Patients at moderate to high risk for developing CIN who were referred for elective CAG or PCI	64	47.5	132.6			18.9		SC vs. SB	Not mentioned	125	2	301	Patients at moderate to high risk for developing CIN who were referred for elective CAG or PCI	64	47.5	132.6			18.9		SC vs SB	Not mentioned	125	2

Motohiro et al. [51]	155	eGFR <60 ml/min/1.73 m^2^ who were undergoing coronary angiography	72.5	69.7	136.6	44.3	55	60		SC vs. SB	Iopamidol	135	3	155	eGFR <60 ml/min/1.73 m^2^ who were undergoing coronary angiography	72.5	69.7	136.6	44.3	55	60		SC vs SB	Iopamidol	135	3

Maioli et al. [52]	300	STEMI undergoing primary PCI	65	25	95.9		42.5	21.7	24	Non vs. SB	Iodixanol	216	3	300	STEMI undergoing primary PCI	65	25	95.9		42.5	21.7	24	Non vs SB	Iodixanol	216	3

Lee et al. [53]	382	Diabetic patients with renal disease (serum creatinine >1.1 mg/dl and eGFR <60 ml/min/1.73 m^2^)	68	70.9	132.6	46		100		SC vs. SB	Iodixanol	116.5	3	382	Diabetic patients with renal disease (serum creatinine >1.1 mg/dl and eGFR <60 ml/min/1.73 m^2^)	68	70.9	132.6	46		100		SC vs SB	Iodixanol	116.5	3

Hafiz et al. [54]	320	Patients with baseline renal insufficiency scheduled to undergo catheterization	73	56.9	141.4			47.2		SC vs. SB	Nonionic, low-osmolar	115	3	320	Patients with baseline renal insufficiency scheduled to undergo catheterization	73	56.9	141.4			47.2		SC vs SB	Nonionic, low-osmolar	115	3

Briguori et al. [55]	292	High-risk patients with an eGFR ≤30 ml/min/1.73 m^2^ and/or a risk score ≥11	76	65.4	158.7	32	47	70.2	28.4	SB vs. RenalGuard	Iodixanol	140	3	292	High-risk patients with an eGFR ≤30 ml/min/1.73 m^2^ and/or a risk score ≥11	76	65.4	158.7	32	47	70.2	28.4	SB vs RenalGuard	Iodixanol	140	3

Wróbel et al. [56]	102	Coronary angiography and/or angioplasty, and had comorbidities that increase the risk of CIN	65.5	56.9	236.4					Oral vs. SC	Loversol	69.5	2	102	Coronary angiography and/or angioplasty, and had comorbidities that increase the risk of CIN	65.5	56.9	236.4					Oral vs SC	Loversol	69.5	2

Vasheghani-Farahani et al. [57]	72	CAG, with SCr 1.5 mg/dL within 2 weeks, having at least 1 of the risk factors	62	79.2	151.2	44.2	36.1	34.7	45.8	0.45 SC vs. SB	Iohexol	117.5	3	72	CAG, with SCr 1.5 mg/dL within 2 weeks, having at least 1 of the risk factors	62	79.2	151.2	44.2	36.1	34.7	45.8	0.45 SC vs SB	Iohexol	117.5	3

Cho et al. [58]	91	Undergoing an elective CAG	78	50.5	123			38.5	17.6	SC vs. SB	Isoversol	128	2	91	Undergoing an elective CAG	78	50.5	123			38.5	17.6	SC vs SB	Isoversol	128	2

Vasheghani-Farahani et al. [59]	265	Serum creatinine level of 1.5 mg/dL or greater undergoing elective CAG	63.3	83	145.4	45.9	51.7	21.5		SC vs. SB	Iohexol	114	5	265	Serum creatinine level of 1.5 mg/dL or greater undergoing elective CAG	63.3	83	145.4	45.9	51.7	21.5		SC vs SB	Iohexol	114	5

Tamura et al. [60]	144	Scheduled for elective CAG or PCI	72.8	87.5	121.1	39.1	57.8	58.3		SC vs. SB	Iohexol	85	3	144	Scheduled for elective CAG or PCI	72.8	87.5	121.1	39.1	57.8	58.3		SC vs SB	Iohexol	85	3

Pakfetrat et al. [61]	192	Undergoing elective CAG or PCI	57.9	61.5	97.2	72.2	50.5	29.7	5.2	SC vs. SB	Iodixanol	65	4	192	Undergoing elective CAG or PCI	57.9	61.5	97.2	72.2	50.5	29.7	5.2	SC vs SB	Iodixanol	65	4

Haase et al. [62]	100	At increased risk of postoperative acute renal dysfunction who were scheduled for elective or urgent cardiac surgery necessitating the use of CPB	71	66	90.7					SC vs. SB	Not mentioned		5	100	At increased risk of postoperative acute renal dysfunction who were scheduled for elective or urgent cardiac surgery necessitating the use of CPB	71	66	90.7					SC vs SB	Not mentioned		5

Budhiraja et al. [63]	187	Nonemergent CAG, baseline serum creatinine >1.0 mg/dL, and availability of serum creatinine values at days 1–3	68		125.8	57.2		30.5		SC vs. SB	Iopromide	199	2	187	Nonemergent CAG, baseline serum creatinine >1.0 mg/dL, and availability of serum creatinine values at days 1–3	68		125.8	57.2		30.5		SC vs SB	Iopromide	199	2

Angoulvant et al. [64]	201	Scheduled for elective CAG, with or without PTCA with a baseline SCr < 140 *μ*mol/L	62	80.6	86.2					Oral vs. SC	Not mentioned	290	3	201	Scheduled for elective CAG, with or without PTCA with a baseline SCr< 140 *μ*mol/L	62	80.6	86.2					Oral vs SC	Not mentioned	290	3

Maioli et al. [65]	502	Undergoing coronary angiographic procedures with estimated creatinine clearance <60 ml/min	74	59	107		46.5	59.1		SC vs. SB	Iodixanol	165	3	502	Undergoing coronary angiographic procedures with estimated creatinine clearance <60 ml/min	74	59	107		46.5	59.1		SC vs SB	Iodixanol	165	3

Chen et al. [66]	660	Myocardial ischemia (angina or positive exercise treadmill) scheduled for PCI, with SCr<1.5 mg/dl	60	85	114.9		54	8		Non vs. SC	Iso-osmolar nonionic	285	2	660	Myocardial ischemia (angina or positive exercise treadmill) scheduled for PCI, with SCr < 1.5 mg/dl	60	85	114.9		54	8		Non vs SC	Iso-osmolar nonionic	285	2

Chen et al. [66]	276	Myocardial ischemia (angina or positive exercise treadmill) scheduled for PCI, with SCr ≥ 1.5 mg/dl	63	82	221		41	22		Non vs. SC	Iso-osmolar nonionic	298	2	276	Myocardial ischemia (angina or positive exercise treadmill) scheduled for PCI, with SCr ≥ 1.5 mg/dl	63	82	221		41	22		Non vs SC	Iso-osmolar nonionic	298	2

Brar et al. [67]	353	Patients with stable renal disease and undergoing CAG	71	63.9	131.7	48	57	44.5	27.2	SC vs. SB	Ioxilan	132	5	353	Patients with stable renal disease and undergoing CAG	71	63.9	131.7	48	57	44.5	27.2	SC vs SB	Ioxilan	132	5

Adolph et al. [68]	145	Stable renal insufficiency and undergoing elective diagnostic or interventional coronary angiography	72.6	77.9	132.6			33.8		SC vs. SB	Iodixanol	140	5	145	Stable renal insufficiency and undergoing elective diagnostic or interventional coronary angiography	72.6	77.9	132.6			33.8		SC vs SB	Iodixanol	140	5

Schmidt et al. [69]	96	CAG	67.6	74	146.7			64.6		SC vs. SB	Optiray	186	2	96	CAG	67.6	74	146.7			64.6		SC vs SB	Optiray	186	2

Ozcan et al. [70]	264	Scheduled for CAG or PCI and had a baseline creatinine level >1.2 mg/dL	69	74.6	122.9			45.1	26.5	SC vs. SB	Ioxaglate	110	2	264	Scheduled for CAG or PCI and had a baseline creatinine level >1.2 mg/dL	69	74.6	122.9			45.1	26.5	SC vs SB	Ioxaglate	110	2

Masuda et al. [71]	59	Scheduled to undergo an emergency coronary angiography or intervention	75	44.1	116.2			30.5		SC vs. SB	Iopamidol	116	3	59	Scheduled to undergo an emergency coronary angiography or intervention	75	44.1	116.2			30.5		SC vs SB	Iopamidol	116	3

Dussol et al. [72]	156	CKD, who were undergoing radiological procedures with CM	65	67.9	204.5	33.1		28.8	16	SC vs. SC + diuresis	Nonionic, low osmolar	117	5	156	CKD, who were undergoing radiological procedures with CM	65	67.9	204.5	33.1		28.8	16	SC vs SC + diuresis	Nonionic, low osmolar	117	5

Mueller et al. [73]	425	Scheduled for elective or emergency PCI	64	75		89		16		0.45 SC vs. SC	Iopromide	226	2	425	Scheduled for elective or emergency PCI	64	75		89		16		0.45 SC vs SC	Iopromide	226	2

Merten et al. [74]	119	Stable renal insufficiency undergoing diagnostic or interventional procedures requiring radiographic contrast, SCr> 1.1 mg/dL	68	74.8	159.1			47.9		SC vs. SB	Iopamidol	132	3	119	Stable renal insufficiency undergoing diagnostic or interventional procedures requiring radiographic contrast, SCr> 1.1 mg/dL	68	74.8	159.1			47.9		SC vs SB	Iopamidol	132	3

Trivedi et al. [75]	53	Scheduled to undergo nonemergency CAG	67.9	98.1	106.4		52.1	18.9		Oral vs. SC	Ionic, low-osmolar	148	2	53	Scheduled to undergo nonemergency CAG	67.9	98.1	106.4		52.1	18.9		Oral vs SC	Ionic, low-osmolar	148	2

Mueller et al. [76]	1383	Scheduled for elective or emergency CAG	64	74.4	81.77			15.7		0.45 SC vs. SC	Ultravist or imeron	234	2	1383	Scheduled for elective or emergency CAG	64	74.4	81.77			15.7		0.45 SC vs SC	Ultravist, or imeron	234	2

CI-AKI: contrast-induced acute kidney injury; SCr: serum creatinine; eGFR: estimated glomerular filtration rate; LVEF: left ventricular ejection fraction; DM: diabetes mellitus; HF: heart failure; CM: contrast media; CTA: computed tomography angiography; TAVI: transcatheter aortic valve implantation; EVAR: elective endovascular aneurysm repair; AAA: abdominal aortic aneurysm; CKD: chronic kidney disease; CRT: cardiac resynchronization therapy; CTPA: computed tomography pulmonary angiography; PE: pulmonary embolism; CIN: contrast-induced nephropathy; CHF: chronic heart failure; CT: computed tomography; TAVR: transcatheter aortic valve replacement; CAG: coronary angiography; CE-CT: contrast media-enhanced computed tomography; CPB: cardiopulmonary bypass; PTCA: percutaneous transluminal coronary angioplasty; NAC: N-acetylcysteine. Treatment groups: SC: intravenous 0.9% sodium chloride; SB: intravenous sodium bicarbonate; Non: nonhydration; Oral: oral hydration; RenalGuard: RenalGuard system; HDy: hemodynamic guided hydration; SC + diuresis: intravenous 0.9% sodium chloride + diuresis; 0.45 SC: 0.45% sodium chloride.

## Abbreviations

CI-AKI: Contrast-induced acute kidney injury
SCr: Serum creatinine
eGFR: Estimated glomerular filtration rate
LVEF: Left ventricular ejection fraction
DM: Diabetes mellitus
HF: Heart failure
CM: Contrast medium
CTA: Computed tomography angiography
TAVI: Transcatheter aortic valve implantation
EVAR: Elective endovascular aneurysm repair
AAA: Abdominal aortic aneurysm
CKD: Chronic kidney disease
CRT: Cardiac resynchronization therapy
CTPA: Computed tomography pulmonary angiography
PE: Pulmonary embolism
CIN: Contrast-induced nephropathy
CHF: Chronic heart failure
CT: Computed tomography
TAVR: Transcatheter aortic valve replacement
CAG: Coronary angiography
CE-CT: Contrast media-enhanced computed tomography
CPB: Cardiopulmonary bypass
PTCA: Percutaneous transluminal coronary angioplasty.
